# Homochiral Carboxylate‐Anchored Truxene Tripods: Design, Synthesis, and Monolayer Formation on Ag(111)

**DOI:** 10.1002/chem.202404750

**Published:** 2025-02-21

**Authors:** Fumitaka Ishiwari, Takuya Omine, Akinori Saeki, Kirsty Munro, Manfred Buck, Michael Zharnikov

**Affiliations:** ^1^ Department of Applied Chemistry Graduate School of Engineering Osaka University Yamadaoka 2-1 Suita Osaka 565-0871 Japan; ^2^ PRESTO Japan Science and Technology Agency (JST) Kawaguchi Saitama 332-0012 Japan; ^3^ Innovative Catalysis Science Division Institute for Open and Transdisciplinary Research Initiatives (ICS-OTRI) Osaka University 1-1 Yamadaoka Suita Osaka 565-0871 Japan; ^4^ EaStCHEM School of Chemistry University of St Andrews North Haugh St Andrews KY16 9ST United Kingdom; ^5^ Angewandte Physikalische Chemie Universität Heidelberg Im Neuenheimer Feld 253 69120 Heidelberg Germany

**Keywords:** molecular tripods, chirality, truxene, scanning probe microscopy, X-ray absorption spectroscopy

## Abstract

The design of well‐defined assemblies of chiral molecules is a prerequisite for numerous applications, such as chirality‐induced spin selectivity (CISS). In this context, tripodal molecular films bear the advantage of better control of molecular orientation and alignment than analogous monopodal systems. To this end, we report on the synthesis and assembly property of *C*_3_ chiral *syn*‐5,10,15‐truxene triacetic acid. (*S*,*S*,*S*) and (*R*,*R*,*R*) enantiomers were isolated and adsorbed on underpotential deposited Ag(111)/Au/mica both individually and as a racemate. The enantiomers form a densely packed and well‐ordered structure (including the azimuthal alignment), even though with small sizes of individual domains. The molecules adsorb predominantly in tripodal configuration, with all three docking groups bound to the substrate as carboxylates in a bidentate fashion. The truxene backbone is then oriented parallel to the substrate surface but the fluorene blades are twisted to some extent. The racemate monolayer turned out to be less densely packed and less well‐ordered compared to the films of individual enantiomers, which underlines the fact that uniform chirality is primarily important for molecular ordering of the truxenes. We hope that the designed system will be useful in the context of CISS and stimulate further activities regarding chiral tripods.

## Graphical abstract

Stimulated by the renewed interest in chirality‐based optical and electronic properties of molecules, a chiral truxene‐based molecular tripod, namely truxene trisacetic acid (TTA), was newly synthesized and chirally resolved. A systematic study using synchrotron‐based spectroscopic techniques and scanning tunneling microscopy revealed that homochiral TTA forms a densely packed and ordered monolayer on the Ag(111) surface.

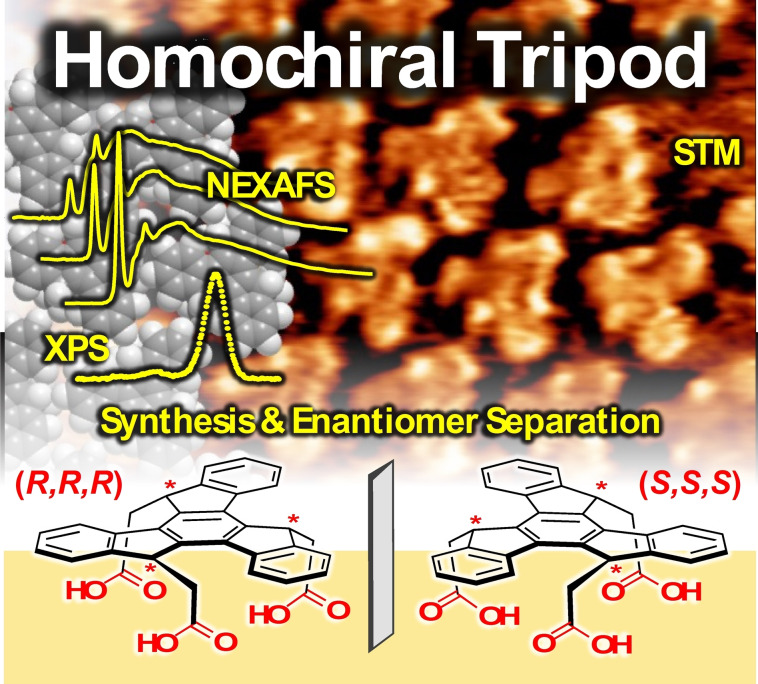

## Introduction

**Figure 1:**
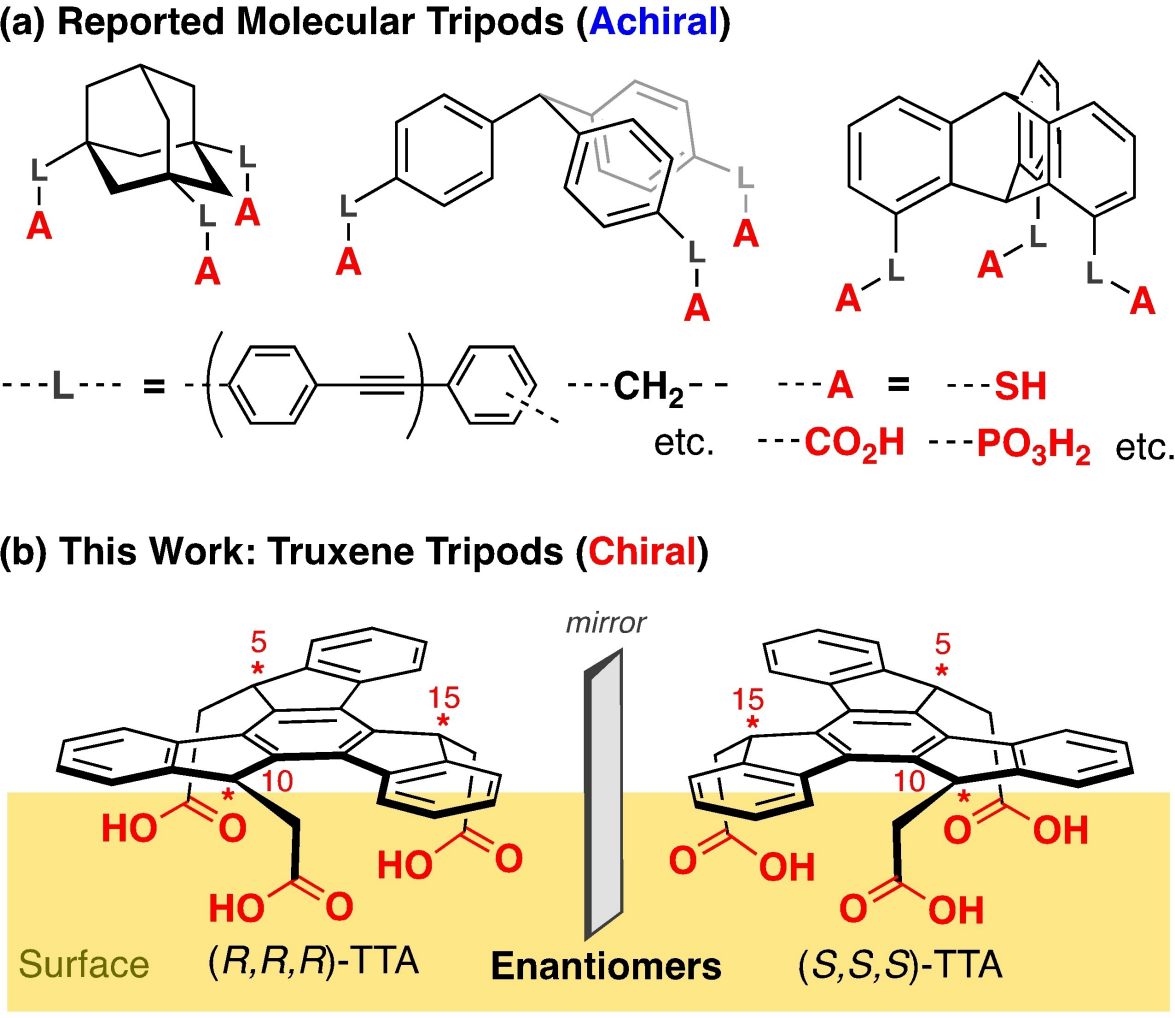
(a) Reported achiral molecular tripods. (b) Chiral (*R,R,R*)‐TTA and (*S,S,S*)‐TTA tripods described in the present work.

Chiral molecules and helical systems have since long been a popular field of research, because of various useful properties and potential applications associated with their chirality,[^1^, ^2^, ^3^, ^4^, ^5^, ^6^] such as chiral recognition ability and circularly polarized luminescence.[^3^, ^4^] As an emerging topic in this context, recent years have seen increasing attention toward chirality‐induced spin selectivity (CISS) of chiral materials.[^7^, ^8^, ^9^, ^10^, ^11^, ^12^, ^13^] Among different classes of chiral molecules, π‐conjugated ones are particularly promising because of their robustness, flexible design, and the possibility of their controlled adsorption on different substrates, including magnetic ones.[^1^, ^2^, ^4^, ^6^, ^13^, ^14^] A promising molecule in this context is truxene (10,15‐dihydro‐5*H*‐diindeno[1,2‐*a*;1’,2’‐c]fluorene) which is a star‐shaped polycyclic aromatic hydrocarbon made up of three fluorene units arranged symmetrically and sharing a common central benzene moiety. Versatile chemical engineering around the truxene scaffold is possible, adapting it to specific applications.^[15]^ This approach allows for the fabrication of dendrimers, oligomers, and polymers useful in the context of photoresists, photoluminescence, organic lasers, and organic electronics and photovoltaics.[^15^, ^16^] Alternatively, the truxene scaffold can be decorated with side or anchoring groups thus enabling its controlled adsorption on a target substrate. Among various substitution motifs, this decoration can be performed such that a tripodal adsorption configuration can be realized which imposes a molecular orientation that can be directly linked to CISS. Previously, various molecular tripod systems based on adamantane, triarylmethane, triarylsilane, and triptycene‐skeletons with thiol, carboxylic acid (CA), and phosphonic acid anchoring groups have been developed (Figure 1a), which, however, are all achiral.[^17^, ^18^, ^19^, ^20^, ^21^] Occasionally, chiral assembly of an achiral tripod on a surface has been observed,^[22]^ but controlling chirality in such systems is difficult.

In the present study, we report on the synthesis and monolayer assembly property of *syn*‐5,10,15‐truxene triacetic acid (TTA) with 5,10,15‐*syn*‐trisubstituted *C*_3_ chiral truxene as a new skeleton (Figure 1b). In this molecular tripod, the sp^3^ carbon atoms at 5, 10, 15 positions of the truxene skeleton are chiral carbons and cause the isolable (*S*,*S*,*S*) and (*R*,*R*,*R*) enantiomers (Figure 1b), abbreviated as (*S*,*S*,*S*)‐TTA and (*R*,*R*,*R*)‐TTA, respectively. So far, only *syn*‐5,10,15‐cyanomethyl truxene was reported and the single molecular surface adsorption behavior on KBr(001) was studied, but this tripod was used as a racemate, and no assembly to an ordered phase was observed.^[23]^

In the present case of (*S*,*S*,*S*)‐TTA and (*R*,*R*,*R*)‐TTA, the choice of CA as the docking group was made because of its affinity to various substrates, both metals, such as silver and copper,^[24]^ and oxides, such as indium tin oxide (ITO) and alumina.[^25^, ^26^] The introduction of the methylene linker between the truxene backbone and the CA groups allows for additional flexibility in the anchoring configuration, particularly important for targeted tripodal adsorption.^[21]^ We selected silver as the test substrate, mimicking an Ag(111) surface by underpotential deposition of a silver bilayer onto an Au(111) support. This approach is well‐established, resulting in high‐quality Ag(111) substrates, suitable for a monomolecular assembly.[^24^, ^27^]

## Results and Discussion

### Synthesis and Enantiomer Separation

**Figure 2:**
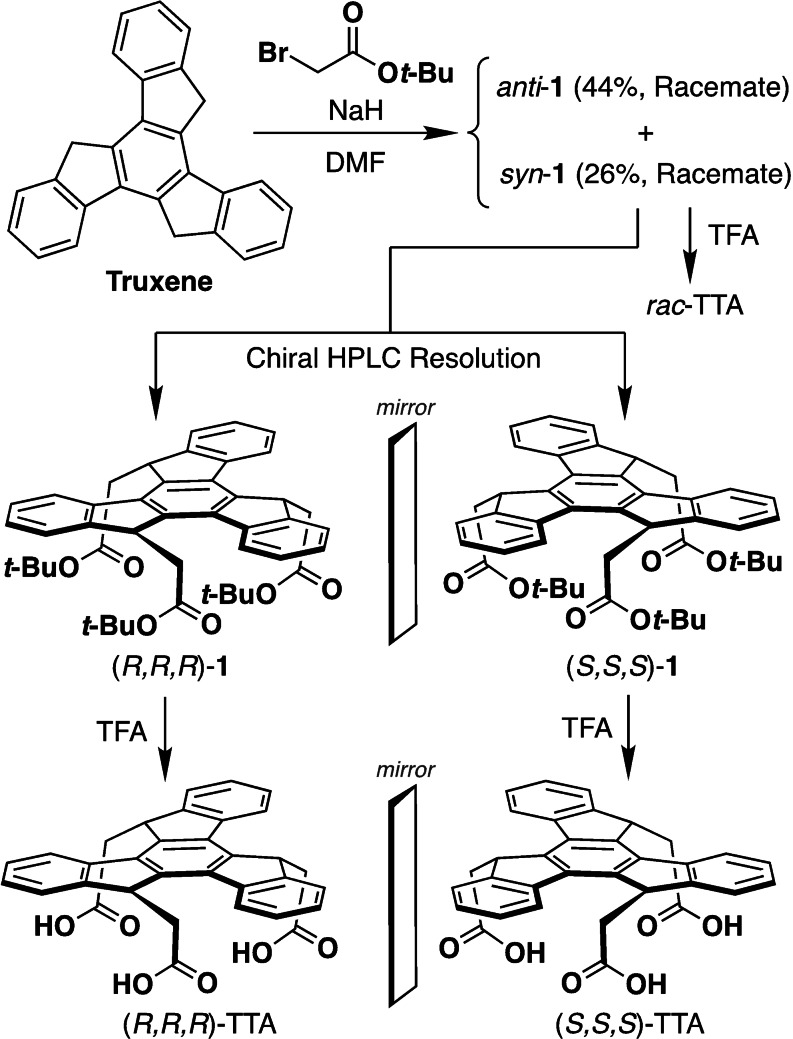
Synthesis scheme of (*R*,*R*,*R*)‐TTA and (*S*,*S*,*S*)‐TTA.

**Figure 3:**
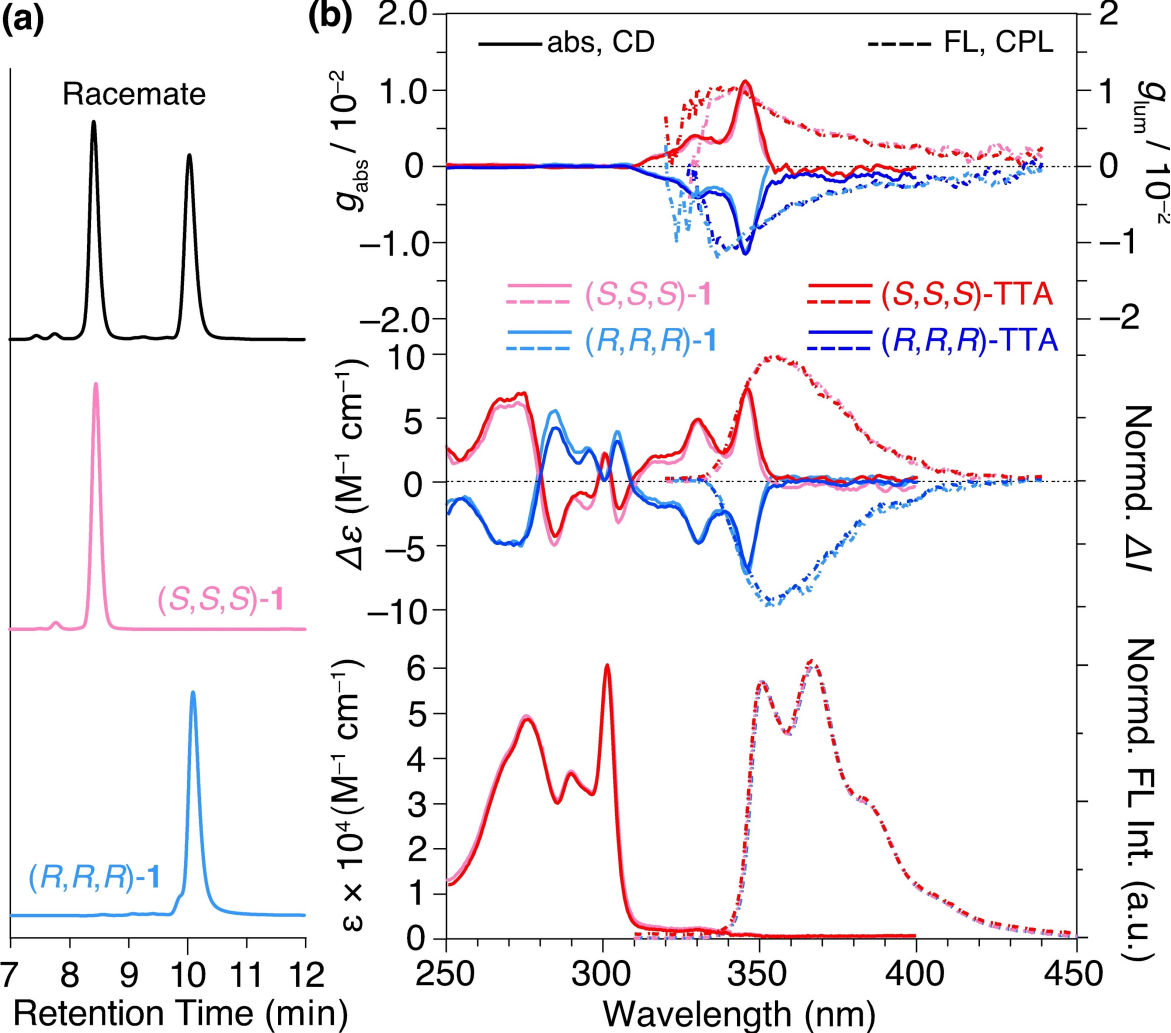
(a) Chiral HPLC traces of racemic *syn*‐**1** (black), enantiopure (*S*,*S*,*S*)‐**1** (pink) and (*R*,*R*,*R*)‐**1** (sky blue) (Daicel Co., Ltd., CHIRAL PAK IF‐3 column, eluent: hexane/CHCl_3_=1/1, v/v; flow rate: 0.5 mL/min; detection: UV absorption at 260 nm). (b) UV‐vis absorption, CD (solid lines), fluorescence and CPL (dotted lines, *λ*_ex_=260 nm) spectra of (*S*,*S*,*S*)‐**1** (pink), (*R*,*R*,*R*)‐**1** (sky blue), (*S*,*S*,*S*)‐TTA (red), (*R*,*R*,*R*)‐TTA (blue) measured in CH_2_Cl_2_ for (*S*,*S*,*S*)‐**1** and (*R*,*R*,*R*)‐**1**, or in tetrahydrofuran for TTAs (15 *μ*M) at 298 K.

The truxene‐based tripods were synthesized according to the scheme in Figure 2 by a procedure similar to our previous report on the synthesis of trisubstituted truxenes.^[28]^ Truxene trianion generated by NaH as a base was reacted with *tert*‐butyl bromoacetate in dimethylformamide (DMF) to give a mixture of *syn*‐**1** [(*S*,*S*,*S*) and (*R*,*R*,*R*) enantiomers] and *anti*‐**1** [(*S*,*S*,*R*) and (*R*,*R*,*S*) enantiomers] as their racemates. *Syn*‐**1** was isolated by SiO_2_‐based medium‐pressure liquid chromatography (MPLC). Enantiomer resolution was performed by chiral HPLC to afford enantiopure (*R*,*R*,*R*)‐**1** and (*S*,*S*,*S*)‐**1**, which was confirmed by chiral HPLC analysis (Figure 3a) and mirror imaged CD and CPL spectra of each enantiomer (Figure 3b, pink and sky blue curves). Because they exhibited almost identical circular dichroism (CD) and circularly polarized luminescence (CPL) spectra with a high dissymmetry factor (*g* value) of 10^–2^ in the order of those of other trisubstituted truxenes,^[28]^ their stereo structures were determined by comparing the spectral shapes and signs of CD and CPL with reported other trisubstituted truxenes.^[28]^ Then the *tert*‐butyl groups of enantiopure (*R*,*R*,*R*)‐**1** and (*S*,*S*,*S*)‐**1** were removed by trifluoroacetic acid (TFA) to give optically active truxene‐based tripods (*R*,*R*,*R*)‐TTA and (*S*,*S*,*S*)‐TTA, respectively. Thus obtained (*R*,*R*,*R*)‐TTA and (*S*,*S*,*S*)‐TTA exhibited almost identical CD spectral shapes and intensity (Figure 3b, red and blue curves) to those of (*R*,*R*,*R*)‐**1** and (*S*,*S*,*S*)‐**1** (Figure 3b, pink and sky blue curves), indicating that (*R*,*R*,*R*)‐TTA and (*S*,*S*,*S*)‐TTA maintain the enantiopurity from the precursors. The racemic tripod (*rac*‐TTA) was directly synthesized from racemic *syn*‐**1** with TFA as a reference compound in the same manner. The chemical structures of the newly synthesized compounds and their intermediates were unambiguously characterized by NMR and FT‐IR spectroscopy, as well as high‐resolution mass spectrometry.

### Adsorption on Ag(111) and Characterization

Thus obtained *rac*‐TTA and (*R*,*R*,*R*)‐TTA were adsorbed from the solution phase on the underpotentially deposited (UPD) Ag(111) substrates on Au(111). The samples were characterized by scanning tunneling microscopy (STM), X‐ray photoelectron spectroscopy (XPS), and near‐edge X‐ray absorption fine structure (NEXAFS) spectroscopy.

#### Scanning Tunneling Microscopy

**Figure 4:**
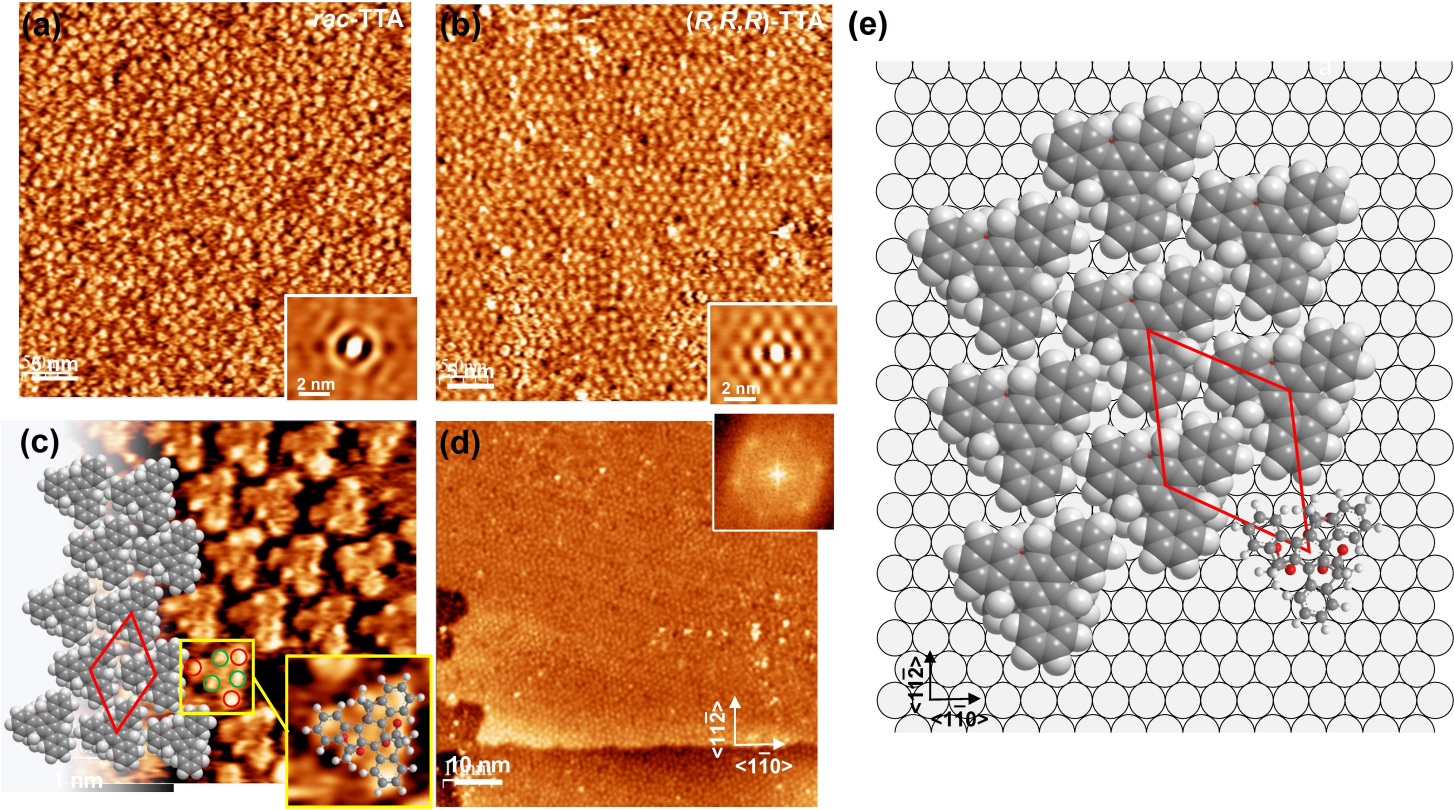
STM images of *rac*‐TTA (a) and (*R*,*R*,*R*)‐TTA (b‐d) monolayers on UPD−Ag/Au/mica with respective autocorrelation images (a,b) and Fourier transform image (d) shown in the insets. (c) High resolution image of the (*R*,*R*,*R*)‐TTA monolayer with the molecular model overlaid. The unit cell is indicated by the red rhombus. (d) (*R*,*R*,*R*)‐TTA monolayer in an area, which includes a monoatomic step in the substrate at the bottom of the image and illustrates that the Fourier transform pattern is rotated by 8° relative to the <1‐10> directions. (e) Model of the (*R*,*R*,*R*)‐TTA monolayer showing the (√19×√19)R8° unit cell in red with an area per molecule of 137.4 Å^2^, corresponding to a coverage of 7.28⋅10^13^ molecules/cm^2^.

STM images of the *rac*‐TTA and (*R*,*R*,*R*)‐TTA monolayers on UPD−Ag/Au/mica, at molecular and submolecular resolution, are presented in Figure 4. Since film formation of the enantiomers is presumably identical, detailed structural studies were performed on only one enantiomer. In both cases, racemate and pure enantiomer, densely packed layers are formed as illustrated by the images in Figures 4a and 4b, but there are pronounced differences as revealed by the autocorrelation images shown in the insets. The racemate film lacks azimuthal alignment and a structural correlation beyond an average nearest neighbour distance of about 1.45 nm. In contrast, the enantiopure layer shows both an azimuthal orientation and a periodic arrangement over several intermolecular distances. Correspondingly, small ordered domains up to about 20 nm in size with predominant, rather anisotropic shapes are seen in the STM images. The small size of these domains can be related to kinetic factors limiting the formation of large domains for such large and specifically shaped molecules as (*R*,*R*,*R*)‐TTA. Within individual domains, the molecules exhibit a hexagonal arrangement and their separation by about 1.26 nm indicates a denser packing compared to the racemate layer. Further insight into the packing of the molecules is obtained by looking at high‐resolution images like the one shown in Figure 4c, where, similar to other work,[^6^, ^14^, ^29^, ^30^] stereochemical characterization was possible. The triangular shape and the uniform orientation of the (*R*,*R*,*R*)‐TTA molecules giving rise to a hexagonal chiral packing are clearly discernible. In addition, there are also submolecular features seen as protrusions. Although exhibiting some variations in intensity, two groups can be identified as highlighted by the red and green circles for the molecule framed by the yellow square. Three are located at the corners of a molecule and the other three are at the edges. As illustrated by the inset of Figure 4c which shows a ball and stick model of (*R*,*R*,*R*)‐TTA this pattern can be mapped to the aromatic rings and the position of the chiral carbon atoms with the hydrogens protruding from the truxene plane. Finally, to infer the orientation of the molecular lattice relative to the substrate, we look at Figure 4d, which shows the (*R*,*R*,*R*)‐TTA monolayer in a region with an <1‐10> orientated step of the substrate. The respective Fourier transform image exhibits a hexagonal pattern of spots which is rotated against the <1‐10> direction by about 8°, i. e. the unit cell vectors do not coincide with the symmetry axes of the substrate. The intermolecular distances and orientation of the molecular rows suggest, as illustrated in Figure 4e, a (√19×√19)R8° unit cell of the (*R*,*R*,*R*)‐TTA monolayer, which means enough space for the molecules to adsorb in a flat‐lying geometry with a tripodal anchoring to the substrate.

#### Spectroscopic Characterization

**Figure 5:**
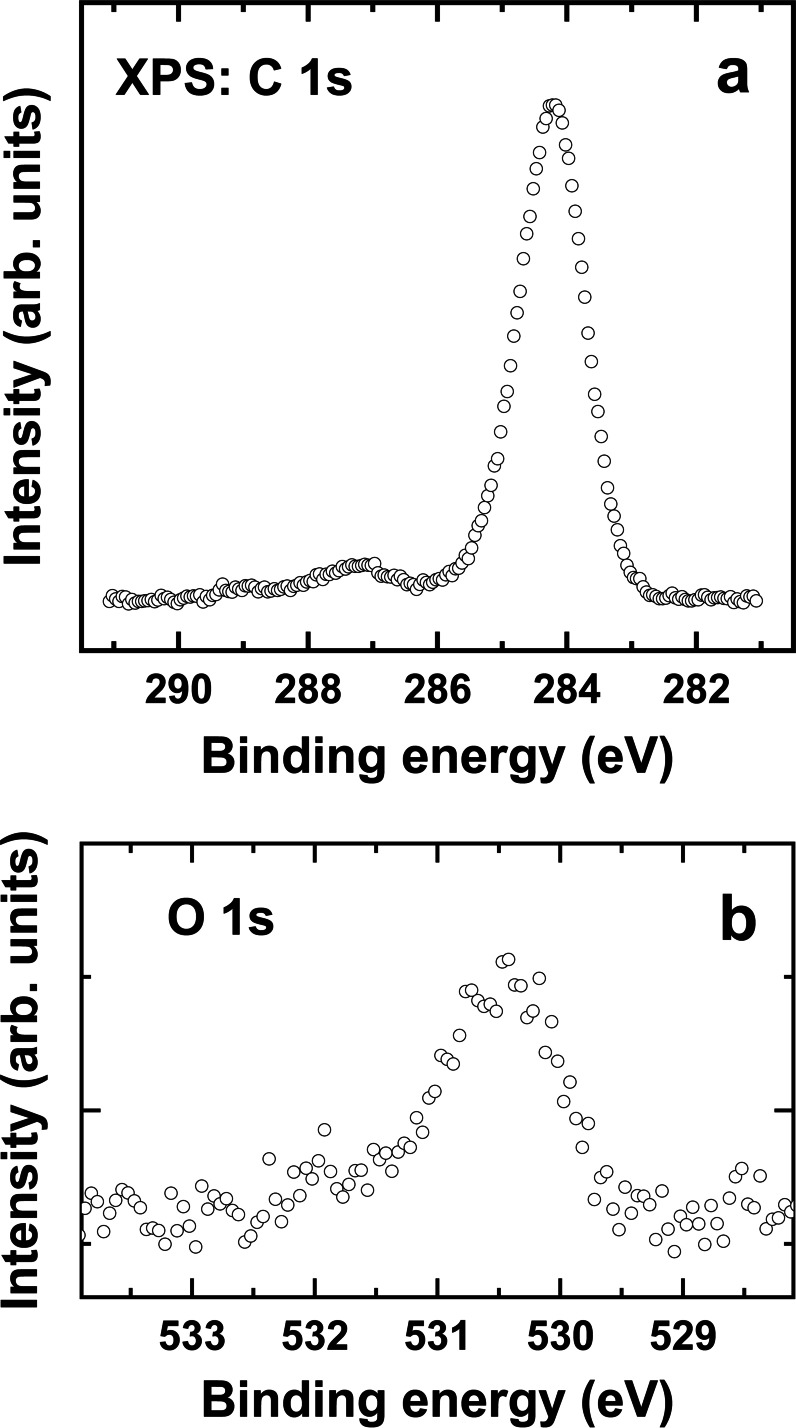
C 1s (a) and O 1s (b) XPS spectra of the (*R*,*R*,*R*)‐TTA monolayer on UPD−Ag/Au/mica. The O 1s spectrum was corrected for the background.

The spectroscopic characterization was only carried out for the (*R*,*R*,*R*)‐TTA monolayer, representative of the (*S*,*S*,*S*)‐TTA case as well, and because of the higher quality of the individual enantiomer films compared to the racemate one (see previous section). The respective XPS data are presented in Figure 5. The C 1s spectrum in Figure 5a is dominated by the strong and symmetric peak at 284.2 eV (typical of aromatic hydrocarbons),^[27]^ corresponding to the truxene framework. This peak is accompanied by a less intense but pronounced feature at 287.2 eV. The respective BE matches the literature value for the carboxylate (COO^–^) group bound to silver[^27^, ^31^] but is distinctly different from that for the unbound COOH group (~288.5 eV)[^27^, ^31^] with only a very weak feature at the respective position. These observations imply the dissociation of hydroxyls in nearly all anchoring CA groups of the (*R*,*R*,*R*)‐TTA molecules and chemisorption of these molecules via symmetrically bonded carboxylates in the tripodal anchoring mode (in agreement with the STM data). The very weak feature at a BE of ~288.5 eV can be related to the limited perfection of this structure (small domains; see previous section) and residual and airborne contamination (C=O; COOH, etc.) associated with domain borders and further structural imperfections.

The above conclusions are supported by the O 1s data (Figure 5b). The respective spectrum shows a single, nearly symmetric peak at 530.45 eV, characteristic of the identical oxygen atoms in the COO^−^ groups[^27^, ^31^] and suggesting chemisorption of nearly all molecules via a symmetrically bonded carboxylate. The features characteristic of the unbound COOH moieties,^[27]^ at ~531.5 eV (carbonyl; C=O) and ~533.5 eV (hydroxyl; C–OH) are hardly perceptible, supporting the hypothesis about the tripodal adsorption of the molecules. There is a weak shoulder at ~532.0 eV which most likely corresponds to residual and airborne contamination, as discussed in relation to the C 1s spectrum.

**Figure 6:**
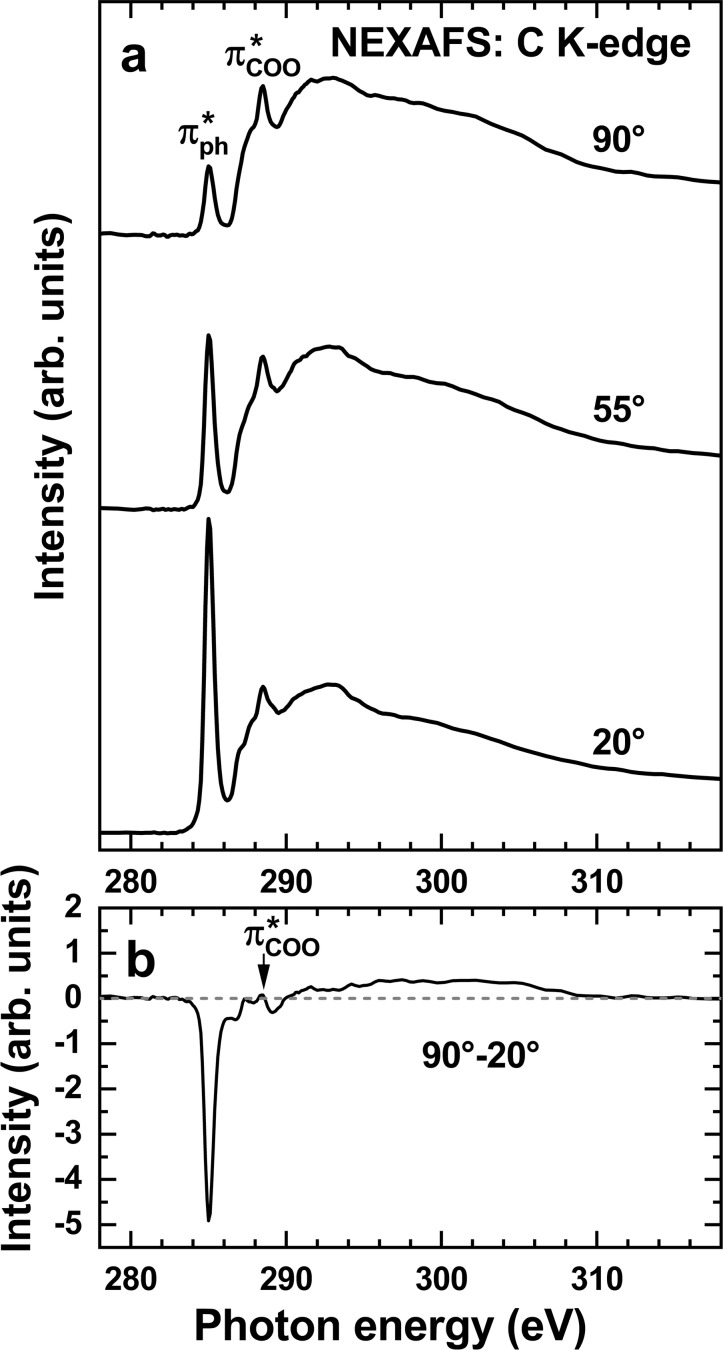
(a) C K‐edge NEXAFS spectra of the (*R*,*R*,*R*)‐TTA monolayer on UPD−Ag/Au/mica acquired at the different X‐ray incidence angles, shown at the spectra. The electric field vector of the synchrotron light, ***E***, is parallel and nearly perpendicular to the substrate at 90° and 20°, respectively. (b) Difference spectrum resulting from subtracting the spectrum acquired at an X‐ray incidence angle of 20° from that acquired at 90°. The most prominent resonances are assigned. The dashed line in (b) corresponds to zero.

Complementary information is provided by the NEXAFS data (Figure 6). The spectra of the (*R*,*R*,*R*)‐TTA monolayer in Figure 6a exhibit the characteristic π*‐type absorption resonances of the molecular building blocks, viz. phenyl[^32^, ^33^] and carboxylate,^[34]^ at 285.0 eV (π_1_*, assigned in the figure as π_ph_*) and 288.5 eV (π_COO_*), respectively. These features are accompanied by the absorption edge and a variety of the σ* resonances at the higher photon energies that are less specific to the individual building blocks of (*R*,*R*,*R*)‐TTA but mostly originate from the truxene backbone. Note that the spectrum of fluorene is dominated by the contributions of the phenylenes.^[33]^

The intensities of the absorption resonances change significantly at the variation of the X‐ray incidence angle, as follows from the original spectra in Figure 6a and emphasized by the difference spectrum in Figure 6b. Specifically, the intensity of the π_1_* resonance of phenyls increases strongly when going from the normal (90°) to grazing (20°) incidence geometry, whereas the intensity of the π_COO_* resonance decreases slightly. The less specific σ* resonances behave similarly to the latter case. Considering that the π* orbitals of the phenylene and carboxylate units are perpendicular to the corresponding building blocks and the σ* orbitals, associated predominantly with the truxene backbone, are parallel to them, this behavior suggests that this backbone is predominantly oriented parallel to the substrate and the carboxylate groups are oriented upright to it.

Considering the small dichroism of the π_COO_* resonance, the average tilt angle of the respective orbitals to the surface normal is just a little lower than the magic angle in NEXAFS spectroscopy, 55°. At this particular orientation, no dichroism is recorded, so a disordered system cannot be distinguished from a fully ordered one.^[32]^ Thus, the planes of the anchoring carboxylate groups, perpendicular to the π_COO_* orbitals, are inclined on average by ca. 30–33° to the surface normal.

**Figure 7:**
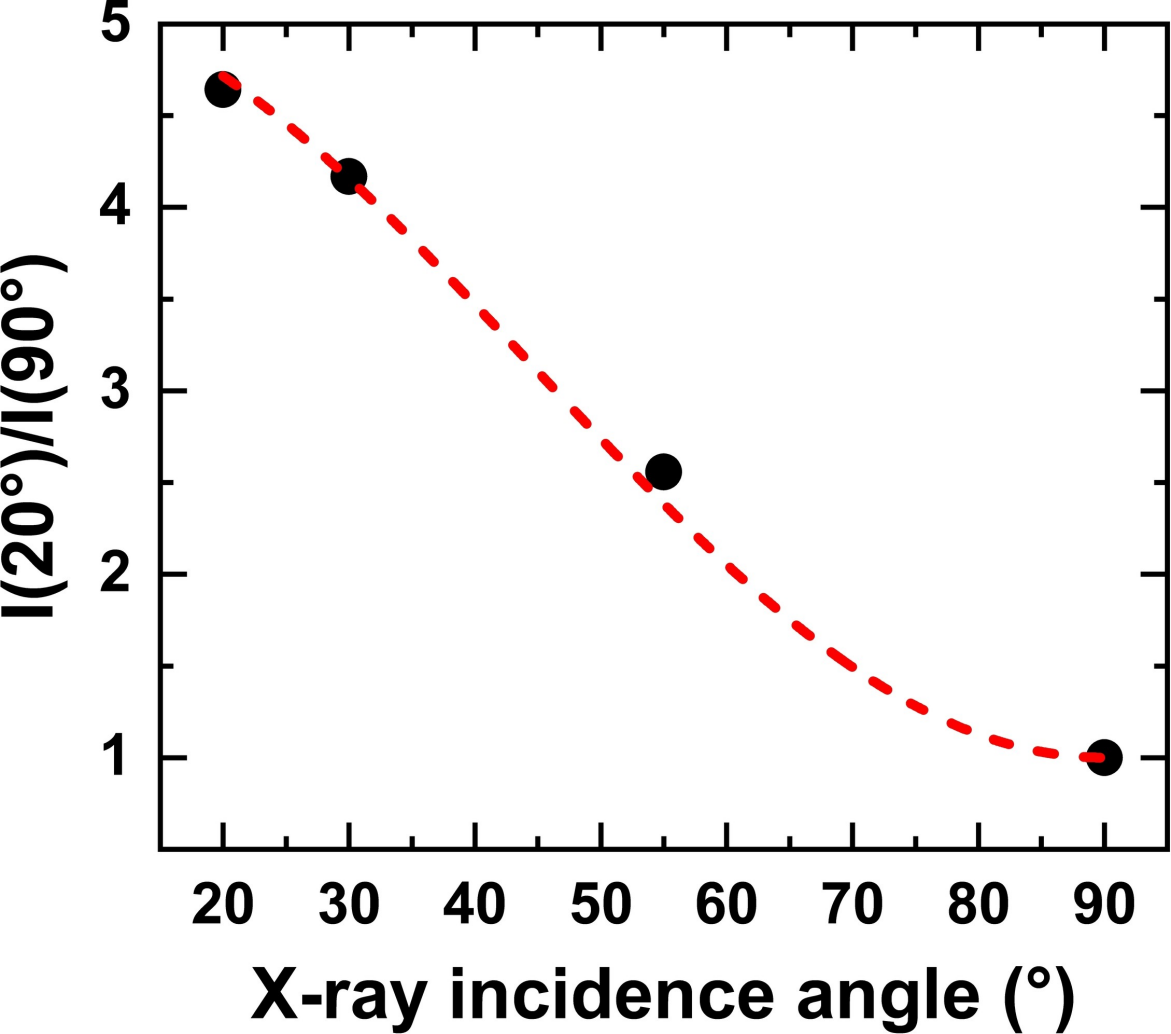
Evaluation of the average tilt of the phenyl rings for the (*R*,*R*,*R*)‐TTA monolayer on UPD−Ag/Au/mica: ratio of the π_1_* resonance intensity in the 20° and 90° spectra as a function of X‐ray incidence angle. The experimental data are shown in black circles; the corresponding fitting curve is drawn in red.

More precise evaluation is possible in the case of phenyl rings, because of the stronger dichroism of the π_1_* resonance. For the threefold or higher system symmetry, characteristic of Ag(111), the intensity of an absorption resonance for the vector orbital, such as π_1_* one, is described by the equation
(1)

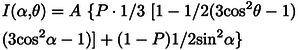




where *A* is a constant, *P* – polarization factor of the primary X‐rays, *θ* – angle between ***E*** and the surface normal (corresponding to the X‐ray incidence angle measured with respect to the substrate surface), and *α* – the average tilt angle of the vector orbital to the surface normal.[^32^, ^35^] The fit of the π_1_* resonance intensities by this equation is shown in Figure 7, with *θ* as the variable and *α* as the fitting parameter.

The resulting value of this parameter is 30±3° to the surface normal, which corresponds to the average tilt angle of the phenyl rings to the surface normal of 60±3° given the perpendicular orientation of the ring plane to the π_1_* orbital. At first sight, this result disagrees with the STM and XPS data which suggest a tripodal adsorption of the molecules, with the truxene backbone parallel to the substrate surface. This contradiction can however be resolved if we assume that the terminal phenyl rings of the fluorene moieties are twisted to some extent with respect to the substrate forced by the asymmetric decoration with the anchoring groups (see Figure 1). Note that a non‐flat conformation is not untypical for substituted truxene^[36]^ and the bonding of the particular enantiomer to the substrate can be accompanied by strain imposed by the anchoring groups and applied asymmetrically to the fluorene moieties. A further factor that contributes to the observed average tilt angle of the phenyl rings in the (*R*,*R*,*R*)‐TTA monolayer is the limited perfection of this monolayer, which includes the small size of individual domains, less ordered areas, and structural defects.

## Conclusions

We have designed and synthesized chiral molecules featuring a truxene backbone decorated with the CA anchoring groups attached to the backbone by methylene linkers. Chirality was introduced by the asymmetric attachment of the anchoring groups to the fluorene blades of truxene. As a prototype of functional, chiral molecular assembly, the adsorption of the (*R*,*R*,*R*)‐TTA enantiomer (representative of the (*S*,*S*,*S*)‐TTA enantiomer as well) and a racemic mixture of both enantiomers on Ag(111) was studied by STM, XPS, and NEXAFS spectroscopy. The (*R*,*R*,*R*)‐TTA enantiomer was found to form densely packed and ordered monomolecular films with a structure commensurate with the underlying substrate and uniform azimuthal alignment of the molecules. The racemate film is less densely packed, lacks crystallinity, and does not show a preferred azimuthal orientation of the molecules.

The (*R*,*R*,*R*)‐TTA molecules were found to adsorb in predominant tripodal geometry, with all three docking groups bound to the substrate as carboxylates, in a bidentate fashion. The truxene backbone is then oriented parallel to the substrate surface but the fluorene blades are tilted to some extent as a consequence of the chirally‐selective attachment of the anchoring groups and their bonding to the substrate. Calculations should be able to reveal to what extent such a twist enhances any chiral properties compared to the undistorted truxene.

The obtained results demonstrate that the CA‐decorated tripodal truxenes can be readily assembled into well‐defined monomolecular films, thus providing a promising platform for chirality‐induced spin‐selective filtering in the near future. Also, introducing suitable coupling groups on the opposite side of the 5,10,15‐positions of the TTA molecule can potentially allow a controlled multilayer deposition via a layer‐by‐layer technique, amplifying the CISS effect.

## Experimental Section

### Materials

Unless otherwise stated, all commercial reagents were used as received. Truxene was prepared according to previously reported procedures^[37]^ and unambiguously characterized by NMR spectroscopy.

### Methods

NMR spectroscopy measurements in solution were carried out on a Bruker AVANCE‐III spectrometer (600 MHz for ^1^H and 150 MHz for ^13^C) or JEOL JNM‐ECS400 (400 MHz for ^1^H and 100 MHz for ^13^C). Chemical shifts (*δ*) are expressed relative to the resonances of the residual non‐deuterated solvents for ^1^H [CDCl_3_: ^1^H(*δ*)=7.26 ppm] and ^13^C [CDCl_3_: ^13^C(*δ*)=77.16 ppm]. Absolute values of the coupling constants are given in Hertz (Hz), regardless of their sign. Multiplicities are abbreviated as singlet (s), doublet (d), triplet (t) and multiplet (m). Infrared (IR) spectra were recorded at 25 °C on a JASCO model FT/IR‐4700AC Fourier‐transform infrared spectrometer. Analytical chiral HPLC was carried out on a JASCO EXTREMA HPLC method scouting system equipped with a JASCO PU‐4180 quaternary pump, a JASCO UV‐4075 UV‐vis detector, a JASCO CD‐4095 CD detector, a JASCO AS‐4050 autosampler and a JASCO CO‐4065 column oven using DAICEL CHIRALPAK^®^ IA‐3, IB N‐3, IC‐3, ID‐3, IE‐3, IF‐3, IG‐3, IH, IJ‐3 and IK columns (diameter: 4.6 mm; length: 250 mm). Recycling preparative chiral HPLC was carried out on a Japan Analytical Industry LC‐7080 Plus recycling preparative HPLC system, equipped with DAICEL CHIRALPAK^®^ IG column (diameter: 20 mm; length: 250 mm). Preparative MPLC was carried out on a Biotage Isolera One MPLC system. High‐resolution matrix‐assisted laser desorption/ionization time of flight (MALDI‐TOF) mass spectrometry measurements were performed on a JEOL model JMS−S3000 mass spectrometer. High‐resolution matrix electro‐ionization (EI) mass spectrometry measurements were performed on a JEOL model JMS‐700EI mass spectrometer. CD spectra were recorded on a JASCO J‐820 AC spectropolarimeter with 10×10 mm cells. CPL spectra were recorded on a JASCO CPL‐300 spectrofluoropolarimeter with 10×10 mm cells under the following conditions: scattering angle: 0°, excitation slit width: 3 mm, emission slit width: 3 mm, scan rate: 100 nm min^–1^, response: 4 sec, accumulation: 8 times; data interval: 0.5 nm, excitation wavelength: 280 nm, HT voltage (photomultiplier): 480 V.

### Synthesis of *syn*‐1

Under nitrogen, a mixture of truxene (500 mg, 1.46 mmol) and 60 % NaH (180 mg, 4.50 mmol) in DMF (60 mL) was sonicated at 4 °C for 1 h, until an almost transparent bright‐red solution had been formed. *tert‐*Butyl bromoacetate (0.74 mL, 5.1 mmol) was then added to the reaction mixture and stirred for 15 min at 4 °C. MeOH (5 mL) was added to the reaction, and the organic solvent was partially evaporated. The residue was dissolved in CH_2_Cl_2_, washed with water and brine, dried over anhydrous MgSO_4_, and evaporated under reduced pressure. The residue was subjected by MPLC (SHOKO SCIENCE, Purif‐Pack‐EX^®^ SI‐50 *μ*m, SIZE‐120, 60 g, hexane/CH_2_Cl_2_=30/70–20/80) to allow isolation of *syn*‐**1** (260 mg, 0.380 mmol, 26 %) and *anti*‐**1** (440 mg, 0.64 mmol, 44 %) as pale‐yellow amorphous. ^1^H NMR (600 MHz, CDCl_3_, Figure S1): *δ* (ppm) 7.99 (d, *J*=7.8 Hz, 3H), 7.68 (d, *J*=7.8 Hz, 3H), 7.45 (dd, *J*=7.8, 7.6 Hz, 3H), 7.37 (dd, *J*=7.8, 7.6 Hz, 3H), 4.90‐4.86 (m, 3H), 3.45‐3.42 (m, 3H), 2.25‐2.19 (m, 3H), 1.56 (s, 27H). ^13^C NMR (151 MHz, CDCl_3_, Figure S2): *δ* (ppm) 171.88, 148.21, 140.14, 139.39, 136.11, 127.75, 126.95, 124.95, 122.68, 81.03, 43.45, 39.02, 28.20. HR EI+ MS: calcd. for C_45_H_48_O_6_ [M]^+^: *m*/*z*=684.3451; found: 684.3445 (Figure S3). FT‐IR (KBr, Figure S4) *ν* (cm^–1^) 3442, 3070, 3046, 2978, 2930, 1730, 1606, 1473, 1458, 1393, 1367, 1350, 1249, 1230, 1144, 1031, 1000, 968, 952, 840, 805, 789, 751, 456. The enantiomer resolution of *syn‐* **1** was performed with Daicel Co., Ltd., CHIRAL PAK IF‐3 column (eluent: hexane/CHCl_3_=1/1, v/v; flow rate: 0.5 mL/min, Figure 3a) to give (*S*,*S*,*S*)‐**1** and (*R*,*R*,*R*)‐**1** as first and second eluents, respectively in >99 enantiopurity.

### Synthesis of *rac*‐TTA, (*S*,*S*,*S*)‐TTA, (*R*,*R*,*R*)‐TTA

Trifluoroacetic acid (5 mL) was added to racemic *syn*‐**1** (100 mg, 0.146 mmol), and the resultant mixture was stirred at 25 °C for 30 minutes and then evaporated under reduced pressure. The residue was washed with hexane and CH_2_Cl_2_ to give *rac*‐TTA (73 mg, 0.141 mmol, 97 %) as white powder. (*S*,*S*,*S*)‐TTA and (*R*,*R*,*R*)‐TTA were obtained with the same procedure from (*S*,*S*,*S*)‐**1** and (*R*,*R*,*R*)‐**1** monomer as white powder in 98 %, and 97 % yields, respectively: ^1^H NMR (600 MHz, Acetone‐*d*_6_, Figure S5): *δ* (ppm) 7.96 (d, *J*=7.5 Hz, 3H), 7.72 (d, *J*=7.5 Hz, 3H), 7.53 (dd, *J*=8.0, 7.5 Hz, 3H), 7.41 (dd, *J*=8.0, 7.5 Hz, 3H), 4.86 (m, 3H), 3.50 (m, 3H), 2.20 (m, 3H). ^13^C NMR (151 MHz, Acetone‐*d*_6_, Figure S6): *δ* (ppm) 172.84, 148.39, 140.11, 139.14, 136.06, 127.86, 127.13, 124.85, 122.54, 42.99, 37.26. HR MALDI‐TOF MS: calced. for C_33_H_24_O_6_Na^+^ [M+Na]^+^: *m*/*z*=539.14651; found: 539.14658 (Figure S7). FT‐IR (KBr, Figure S8) *ν* (cm^–1^) 3423, 3046, 2924, 2653, 2566, 1707, 1472, 1395, 1323, 1275, 1231, 1152, 743, 591, 472.

### Preparation of Ag(111) Substrate

Ag(111) substrates were prepared by underpotential deposition (UPD) on Au(111) templates. These templates (300 nm epitaxial Au(111) layer on mica, Georg Albert PVD, Silz, Germany) were annealed using a natural gas flame before UPD of a pseudomorphic (1×1) Ag bilayer. To this end, Au/mica was immersed in 10 mM AgNO_3_ in 100 mM HNO_3_ (aq) and a potential of 10 mV (vs. Ag/Ag^+^) was applied to the substrate for 2 minutes. This yields full coverage of a stable bilayer of silver atoms.[^38^, ^39^, ^40^, ^41^]

### Adsorption of TTAs on Ag(111)

For SAM formation, substrates were immersed in an aqueous or H_2_O/EtOH truxene solution, using either a particular enantiomer or their mixture. Concentration, temperature, and immersion time were varied but the best results, presented in the given article, were obtained for a 50 μM aqueous solution, a temperature of 70 °C, and an immersion time of 10 min. After immersion, the samples were rinsed with EtOH and blown dry with N_2_.

### STM Experiments

STM imaging was carried out using a Molecular Imaging PicoSPM system in an ambient atmosphere. Tips were mechanically cut from Pt/Ir 80 : 20 wire (Advent Research Materials Ltd, 0.25 mm diameter). Typical parameters of a tunnelling current of 5–10 pA and a tip bias of 0.4‐0.5 V were used. The imaging stability and quality varied substantially from sample to sample but molecular‐resolution imaging was nevertheless possible.

### Spectroscopic Characterization

XPS and NEXAFS experiments were carried out at the bending magnet HE‐SGM beamline of the synchrotron storage ring BESSY II in Berlin, Germany. This beamline provides a linearly polarized synchrotron light with a polarization factor of ~90 %. A custom‐designed experimental station was used.^[42]^ The XPS spectra were collected with a Scienta R3000 electron energy analyzer, in normal emission geometry. The primary photon energy (PE) was set to 580 eV and the energy resolution was ~0.5 eV. The binding energy (BE) scale of the spectra was referenced to the Au 4f_7/2_ emission of the Au substrate at 84.0 eV.^[43]^

The NEXAFS spectra were collected at the carbon K‐edge in the partial electron yield (PEY) mode. The retarding voltage was set to ‐150 V. The incidence angle of the primary X‐ray beam, defined with respect to the sample surface, was varied between the normal (90°; ***E*** vector parallel to the sample surface) and grazing (20°; ***E*** vector nearly perpendicular to the sample surface) incidence geometry to monitor a possible variation in the intensity of the characteristic absorption resonances. This variation, described as the linear dichroism,^[32]^ reflects the molecular orientation in the SAMs.^[35]^ The energy resolution was ~0.3 eV. The PE scale was referenced to the pronounced π* resonance of highly oriented pyrolytic graphite at 285.38 eV.^[44]^ The raw spectra were corrected for the PE dependence of the incident photon flux and reduced to the standard form with zero intensity in the pre‐edge region and the unity jump in the far post‐edge region.

## Conflict of Interests

The authors declare no conflict of interest.

1

## Supporting information

As a service to our authors and readers, this journal provides supporting information supplied by the authors. Such materials are peer reviewed and may be re‐organized for online delivery, but are not copy‐edited or typeset. Technical support issues arising from supporting information (other than missing files) should be addressed to the authors.

Supporting Information

## Data Availability

The data that support the findings of this study are available from the corresponding author upon reasonable request.
