# Supplementary material for: Homochiral Carboxylate‐Anchored Truxene Tripods: Design, Synthesis, and Monolayer Formation on Ag(111)
Source: Chemistry. 2025 Feb 21;31(20):e202404750. doi: 10.1002/chem.202404750 (PMC11973864; doi:10.1002/chem.202404750)
Supplement: Supplementary file 1 — Supporting Information [file CHEM-31-e202404750-s001.pdf]

Chemistry–A European Journal

Supporting Information

Homochiral Carboxylate-Anchored Truxene Tripods: Design, Synthesis, and Monolayer Formation on Ag(111)

Fumitaka Ishiwari,* Takuya Omine, Akinori Saeki, Kirsty Munro, Manfred Buck,* and Michael Zharnikov*

Supporting Information

Homochiral Carboxylate-Anchored Truxene Tripods: Design, Synthesis, and Monolayer Formation on Ag(111)

Fumitaka Ishiwari,*[a,b,c] Takuya Omine,[a] Akinori Saeki,[a,c] Kirsty Munro,[d] Manfred Buck,*[d] and Michael Zharnikov*[e]

[a] *Department of Applied Chemistry, Graduate School of Engineering, Osaka University, 2-1 Yamadaoka, Suita, Osaka 565-0871, Japan*

[b] *PRESTO, Japan Science and Technology Agency (JST), Kawaguchi, Saitama 332-0012, Japan*

[c] *Innovative Catalysis Science Division, Institute for Open and Transdisciplinary Research Initiatives (ICS-OTRI), Osaka University, 1-1 Yamadaoka, Suita, Osaka 565-0871, Japan*

[d] *EaStCHEM School of Chemistry, University of St Andrews, North Haugh, St Andrews KY16 9ST, United Kingdom*

[e] *Angewandte Physikalische Chemie, Universität Heidelberg, Im Neuenheimer Feld 253, 69120 Heidelberg, Germany*

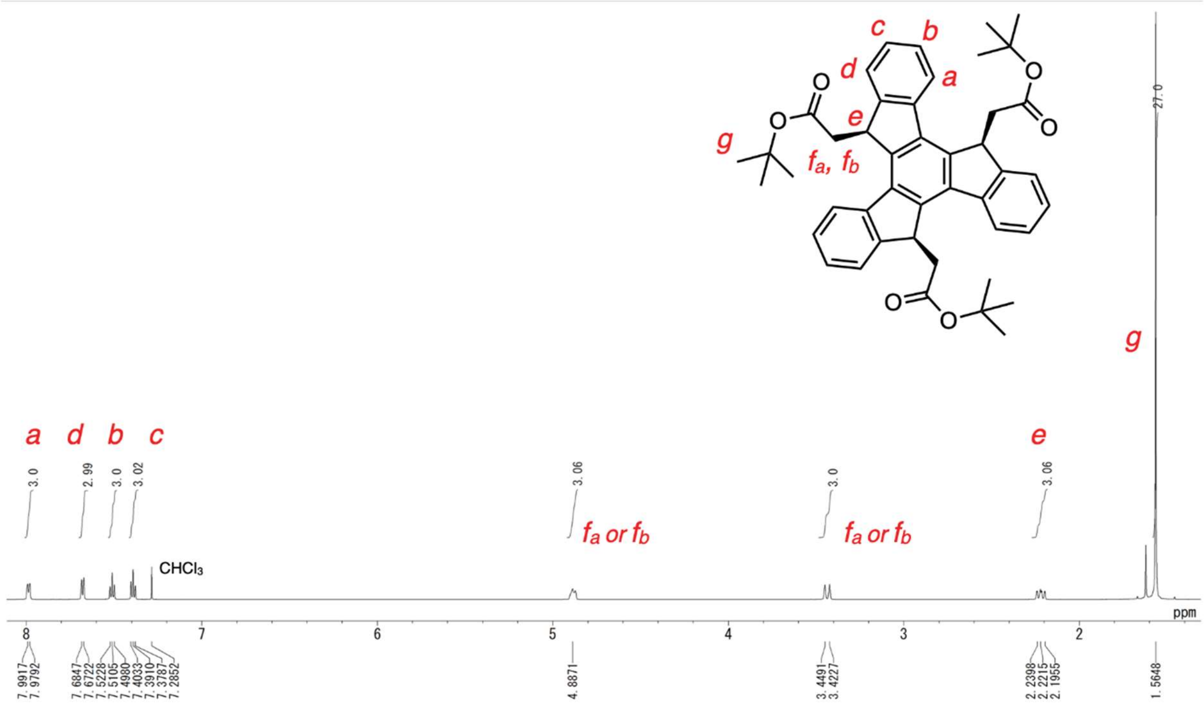

Figure S1. ^{1}H NMR spectrum (600 MHz, $CDCl_3$) of racemic *syn*-**1**.

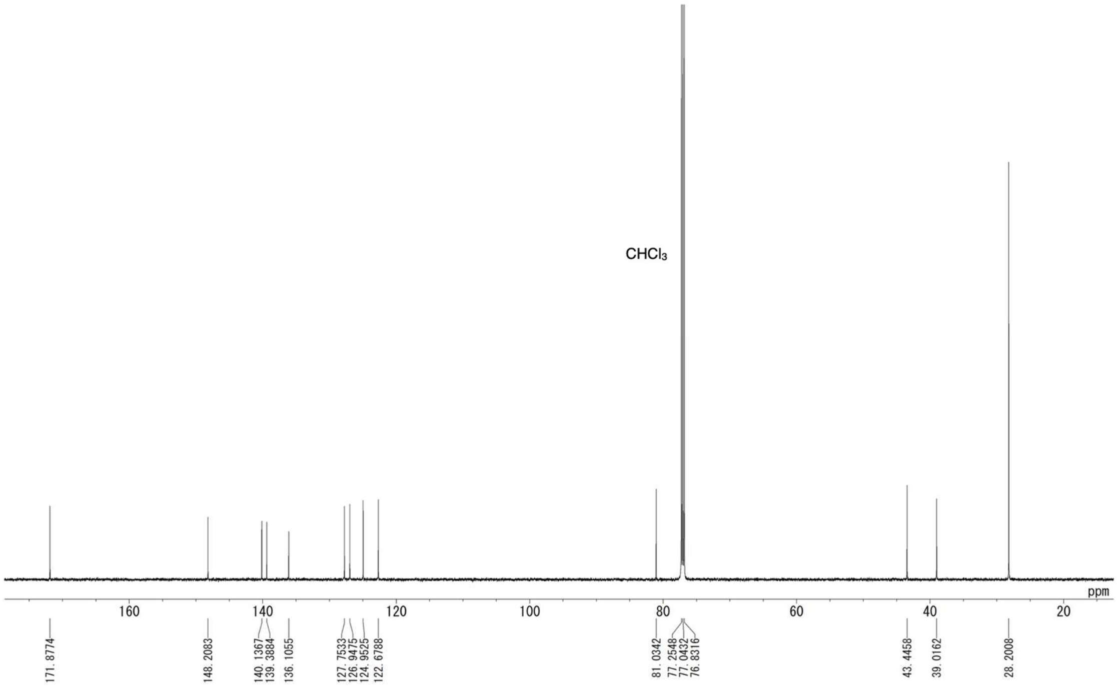

Figure S2. ^{13}C NMR spectrum (151 MHz, $CDCl_3$) of racemic *syn*-**1**.

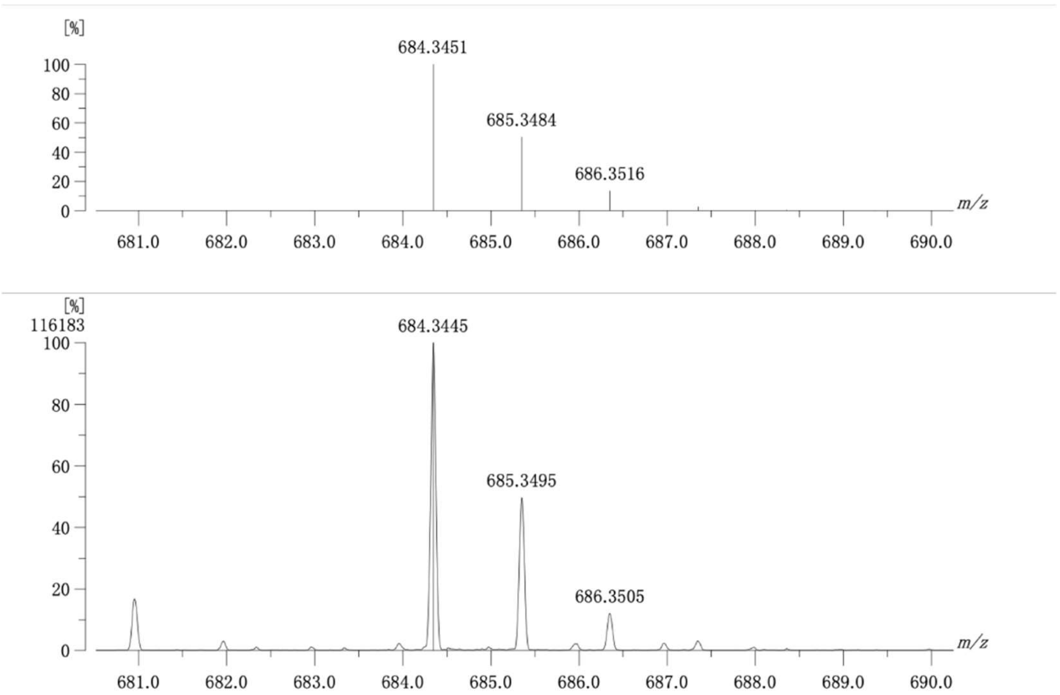

Figure S3. Simulated (upper) and observed (lower) high-resolution EI^+ mass spectra of racemic *syn*-**1**.

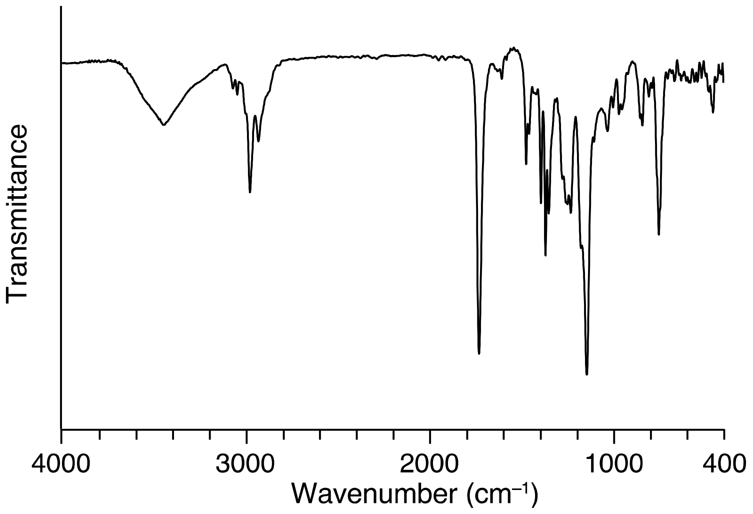

Figure S4. FT-IR spectrum of racemic *syn*-**1** (KBr).

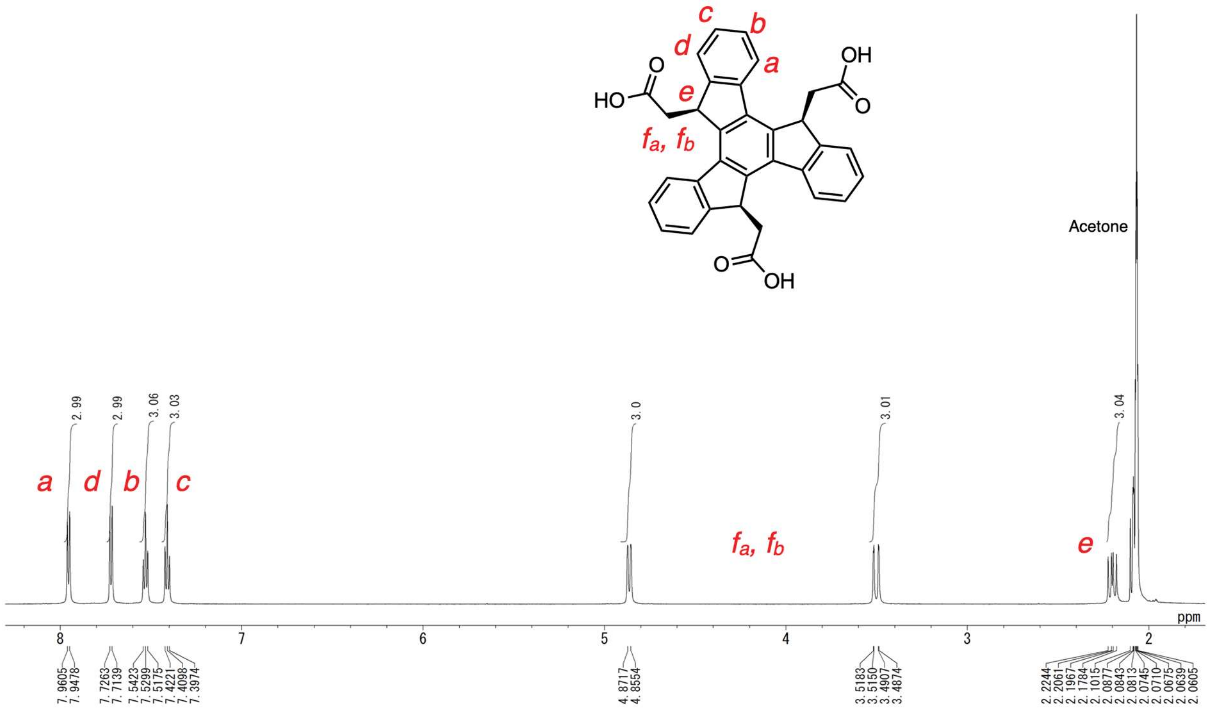

Figure S5. ^{1}H NMR spectrum (600 MHz, Acetone-d_6) of racemic **TTA**.

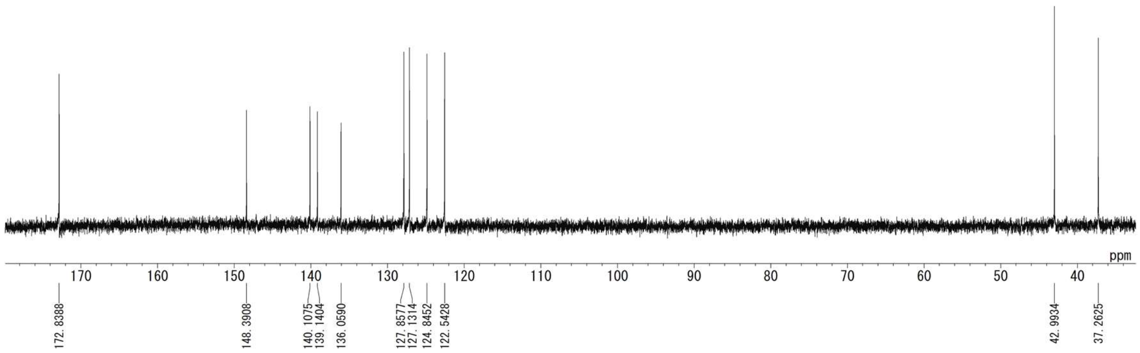

Figure S6. ^{13}C NMR spectrum (151 MHz, Acetone-d_6) of racemic **TTA**.

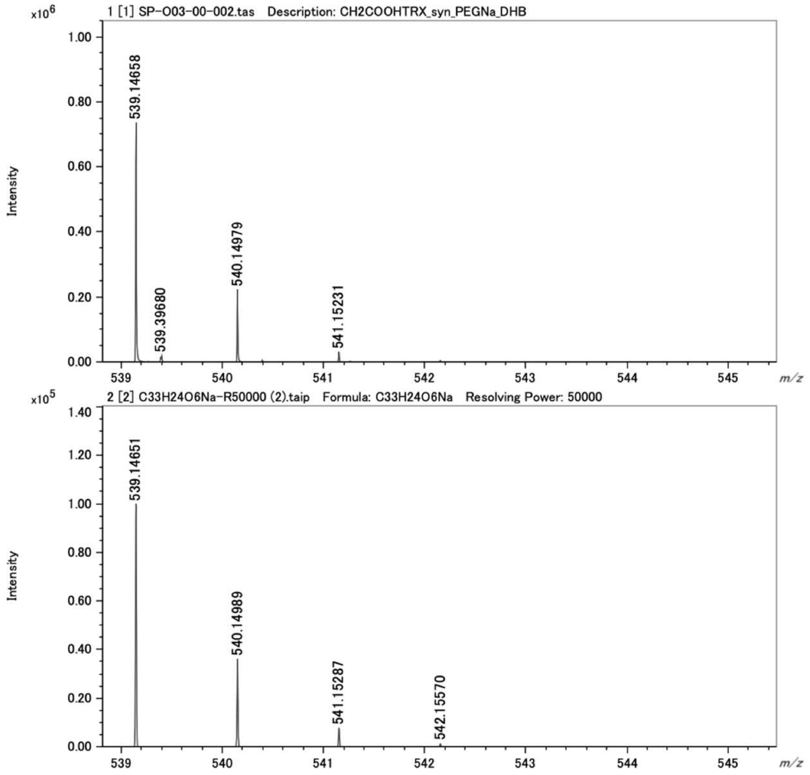

Figure S7. Observed (upper) and simulated (lower) high-resolution MALDI-TOF mass spectra of racemic **TTA**.

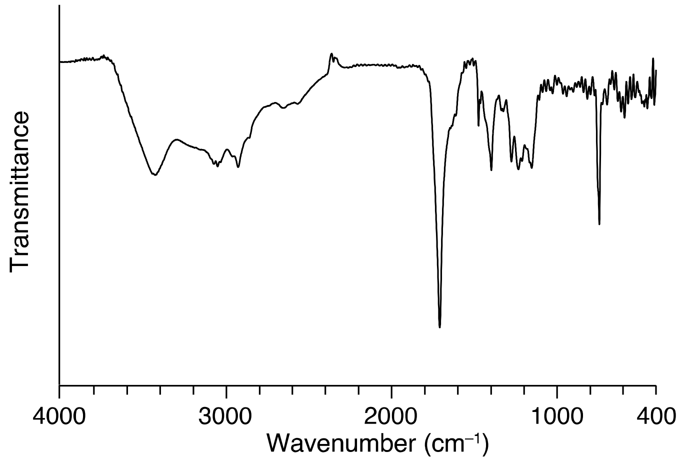

Figure S8. FT-IR spectrum of racemic **TTA** (KBr).
